# Advances in targeting histone deacetylase for treatment of solid tumors

**DOI:** 10.1186/s13045-024-01551-8

**Published:** 2024-05-31

**Authors:** Mu-Qi Shi, Ying Xu, Xin Fu, De-Si Pan, Xian-Ping Lu, Yi Xiao, Yi-Zhou Jiang

**Affiliations:** 1 Department of Breast Surgery, Fudan University Shanghai Cancer Center, Shanghai, 200032 China; 2 Department of Oncology, Shanghai Medical College, Fudan University, Shanghai, 200032 China; 3 Shenzhen Chipscreen Biosciences Co., Ltd., Shenzhen, 518055 People’s Republic of China

**Keywords:** Histone deacetylase, Histone deacetylase inhibitor, Solid tumor, Combination therapy, Clinical administration

## Abstract

Histone deacetylase (HDAC) serves as a critical molecular regulator in the pathobiology of various malignancies and have garnered attention as a viable target for therapeutic intervention. A variety of HDAC inhibitors (HDACis) have been developed to target HDACs. Many preclinical studies have conclusively demonstrated the antitumor effects of HDACis, whether used as monotherapy or in combination treatments. On this basis, researchers have conducted various clinical studies to evaluate the potential of selective and pan-HDACis in clinical settings. In our work, we extensively summarized and organized current clinical trials, providing a comprehensive overview of the current clinical advancements in targeting HDAC therapy. Furthermore, we engaged in discussions about several clinical trials that did not yield positive outcomes, analyzing the factors that led to their lack of anticipated therapeutic effectiveness. Apart from the experimental design factors, issues such as toxicological side effects, tumor heterogeneity, and unexpected off-target effects also contributed to these less-than-expected results. These challenges have naturally become significant barriers to the application of HDACis. Despite these challenges, we believe that advancements in HDACi research and improvements in combination therapies will pave the way or lead to a broad and hopeful future in the treatment of solid tumors.

## Introduction

Histone acetylation holds a significant position among various histone posttranslational modifications. Since the initial elucidation in 1964, it has been identified to play an indispensable role in an extensive range of DNA-mediated cellular events [1]. The homeostasis of histone acetylation is governed by the dynamic equilibrium between histone deacetylase (HDAC) and histone acetyltransferase (HAT) activities [2]. Intriguingly, in specific solid tumors, aberrant upregulation of HDAC expression perturbs this regulatory balance, which promotes the growth of solid tumors. The HDAC enzyme family has gained recognition for the significant role this family plays in the pathogenesis and progression of these solid tumors [3].

In the relentless pursuit to correct the imbalances seen in tumors with high HDAC expression, researchers have developed an exceptionally effective and practical strategy. This led to the development of a novel class of antitumor therapeutics called HDAC inhibitors (HDACis) [4]. As reported, the strategic inhibition of HDAC activity is promising for curtailing the proliferation and differentiation of neoplastic cells, instigating tumor cell apoptosis, and attenuating tumor-associated angiogenesis [5]. Presently, the United States Food and Drug Administration (FDA) and the National Medical Products Administration (NMPA) of China have granted approval to several HDACis [6, 7]. While these drugs have shown promising results in treating hematological malignancies, their efficacy as standalone treatments for solid tumors often falls short. For example, we have observed limitations in the effectiveness of HDACis in one such case involving urothelial carcinomas [8]. Therefore, there is an urgent need to formulate HDACi-based combination therapy strategies or to design more selective HDACis to improve the efficacy of HDACis in solid tumors.

This review begins by focusing primarily on the basic concept of HDAC and its inhibitors. It then moves on to provide an overview of representative preclinical instances in which combined HDACi therapies have been utilized, delving into clinical trials that have incorporated HDACis for the treatment of solid tumors. Furthermore, it evaluates the overall therapeutic outcomes and current challenges in treating solid tumors, while also offering insights into the future potential of HDACi applications in this area.

## Functions and classification of HDACs

HDACs play a pivotal role in regulating histone acetylation and modulating various signaling pathways. However, their functions exhibit distinct variations depending on their specific classifications. At present, 18 isoforms of HDACs have been found in humans and are categorized into 4 classes (I, II, III and IV) based on their homology to yeast proteins [9]. The human enzymes HDAC1, HDAC2, HDAC3, and HDAC8 are classified as Class I HDACs, and these nuclear proteins exhibit homology to the yeast protein Rpd3 [10, 11]. Class I HDACs work in the form of multiprotein complexes, such as NuRD, Sin3, and CoREST, and are implicated in various physiological processes, including transcriptional repression, chromatin assembly, cell cycle progression, and maintenance of genomic stability [12]. Class II HDACs, including HDAC4, 5, 6, 7, 9, and 10, share homology with the yeast deacetylase Hda1 [13]. Class II HDACs are present in both the nucleus and cytosol, where phosphorylation controls their movement between these locations. The functions of these HDACs are linked to cellular processes such as inflammation and migration [14]. HDAC11 is the only member of Class IV HDAC, demonstrating homology with RPD3 and HDA1 and displaying characteristics of both class I and II HDACs [15, 16]. All 11 isoforms above, encompassing Classes I, II, and IV, are Zn^2+^-dependent proteins [17]. The activation of catalysis occurs through a general acid‒base mechanism utilizing metal-water as the nucleophile. Unlike the previously mentioned three classes, Class III HDACs, which include SIRT1, 2, 3, 4, 5, 6, and 7, constitute a category of NAD^+^-dependent HDACs that exhibit a strict reliance on NAD^+^ and do not require zinc for their functionality [18, 19]. Class III HDACs, exemplified by SIRT2, play a multifaceted role in orchestrating a wide array of physiological and pathological processes, including but not limited to metabolism, regulation of genetic material, and regulation of the cell cycle [20–22]. In general, HDACs exhibit distinct physiological functions and structural characteristics based on their respective classifications (Table 1) [23–26].

**Table 1:** Comparison among the Classes of HDACs

Feature	Class I	Class II	Class III	Class IV
Isoforms	HDAC1, HDAC2, HDAC3, HDAC8	Class IIa: HDAC4, HDAC5, HDAC7, HDAC9; Class IIb: HDAC6, HDAC10	SIRT1–7	HDAC11
Cellular Localization	Nucleus	Nucleus and Cytoplasm	Cytoplasm	Nucleus and Cytoplasm
Cofactors	Zn^2^^+^	Zn^2^^+^	NAD^+^	Zn^2^^+^
Biological functions	Transcriptional regulation, cell cycle control, and apoptosis	Class IIa: Regulate transcription, development; Class IIb: microtubule dynamics, cell motility, and autophagy	Metabolism, stress response, DNA repair, aging, and mitochondrial functions	Protein interactions and signaling pathways
Homology to yeast HDAC enzymes	Rpd3	Hda1	Sir2	/

## HDACs affect tumor progression

Histones undergo dynamic modifications through the action of specific enzymes [27]. The enzymes responsible for adding modifications to histones are referred to as writers, while those responsible for removing modifications are known as erasers [28]. Additionally, enzymes that recognize these modifications are referred to as readers and may recruit other factors to stabilize the chromatin signature [29].

**Fig. 1:**
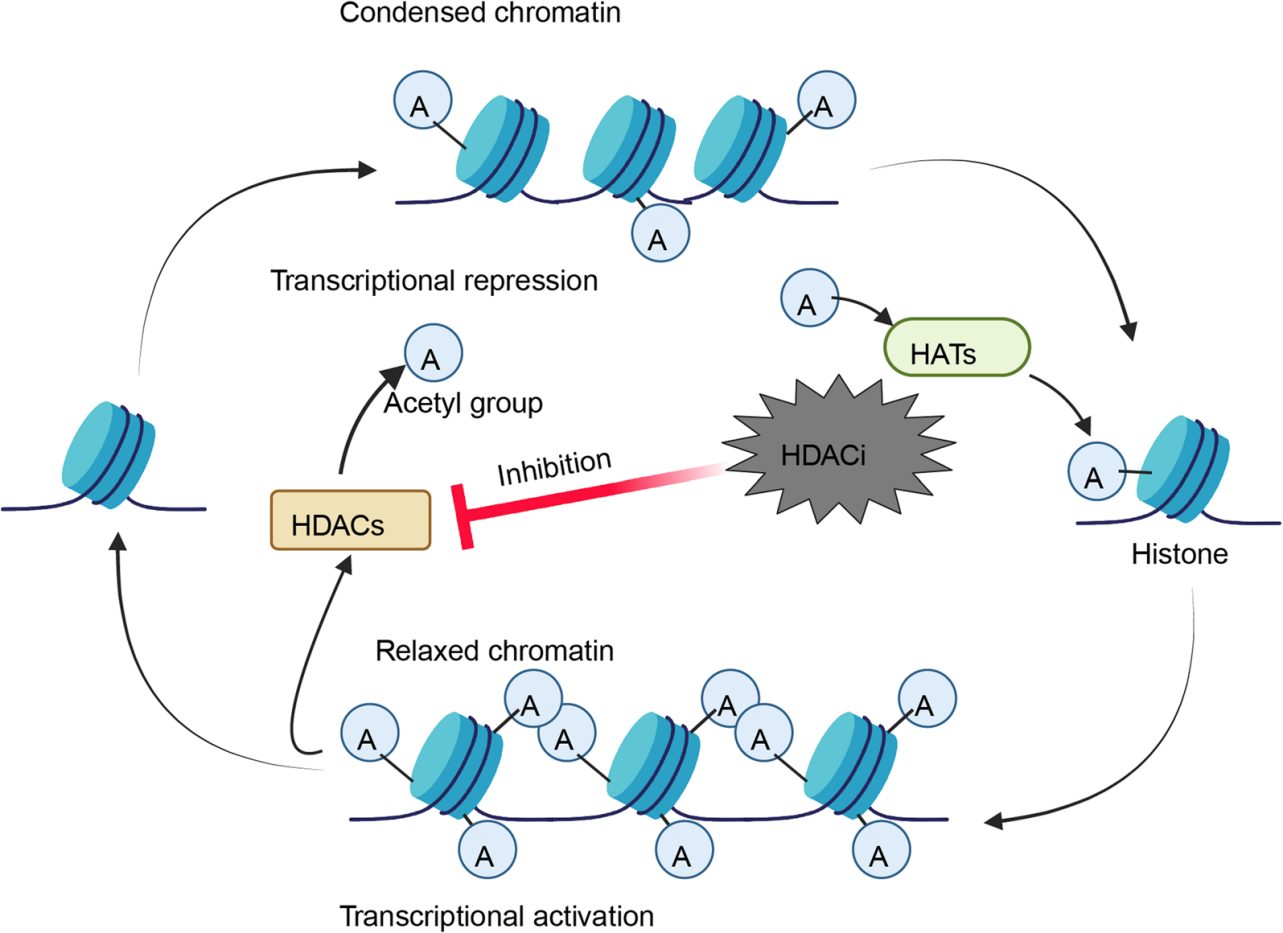
HDACis affect chromatin remodeling and restore the balance between HAT and HDAC. The employment of HDACis leads to a predominant rise in histone acetylation, creating a more accessible and relaxed chromatin structure that is conducive to transcriptional activation, as opposed to the transcriptionally dormant state of densely packed chromatin. Created with BioRender.com

The expression of HDAC is intricately linked with the onset and progression of certain solid tumors. The process of acetylation facilitated by HATs and the process of deacetylation facilitated by HDACs are a set of opposing enzymes involved in the modification of histone acetylation, playing an important role in regulating gene expression (Fig. 1) [30, 31]. In histone acetylations, HAT is responsible for adding acetyl groups to histones, which is typically associated with gene activation and transcription. Thus, HAT can be considered a "writer". HDAC is responsible for removing acetyl groups from histones, which is usually linked to gene silencing and transcriptional repression. Therefore, HDAC can be viewed as an "eraser". A hallmark feature of some types of cancer is the disruption of the balance between HDACs and HATs, manifesting as an elevated level of histone deacetylation [32, 33]. Elevated levels of HDACs contribute to a significant decrease in histone acetylation. This leads to a more compact chromatin structure that is unfavorable for transcriptional activation (Fig. 1) [34]. As a result, the typical expression of specific genes at their respective DNA-binding sites is suppressed. When elevated levels of HDAC lead to the transcriptional repression of specific genes, particularly those that function as tumor suppressors or are involved in other antitumor mechanisms, it can be empirically deduced that high HDAC expression indirectly contributes to both the initiation and progression of tumors [35].

Specifically, HDAC, serving as a pivotal regulatory factor in tumor-associated physiological processes, exerts varying degrees of influence on these tumor-related essential physiological events through transcriptional regulation, encompassing angiogenesis, cell cycle regulation, immunity, DNA repair, and apoptosis. In terms of angiogenesis, HDAC1 orchestrates endothelial cell cycle progression by directly modulating the transcription of cyclin-dependent kinase (CDK) and cyclin genes [36]. Regarding cell cycle regulation, pRB/HDAC complexes govern the expression of the cyclin E gene, and in turn, cyclin E/Cdk2 plays a pivotal role in the regulation of histone gene expression [37–39]. In the realm of immune modulation, HDAC5 curtails the transcriptional activity of p65, thereby influencing PD-L1 expression and cancer immunity [40]. Regarding apoptosis regulation, it has been reported that HDAC oversees the transcription of miR-15 and miR-16, subsequently impacting pancreatic stellate cell apoptosis [41]. While the DNA damage response is not directly associated with transcriptional regulation of HDACs, it is influenced by the acetylation status of histone and nonhistone proteins and by the balancing activities of HAT and HDAC enzymes [42].

In summary, HDAC serves as a hub regulating a diverse spectrum of physiological and pathological processes and promotes tumor growth through histone-dependent or histone-independent mechanisms. As a therapeutic approach to address relevant tumors, HDACis can comprehensively correct the imbalances between HDAC and HAT induced by HDAC overexpression (Fig. 1), thereby delivering antitumor effects on tumors exhibiting elevated HDAC expression via various mechanisms. The effects of aberrant HDAC activity or overexpression may be reversed by the use of HDACis, providing a potential treatment for tumors with high HDAC expression.

## Overview of HDAC inhibitors

**Fig. 2:**
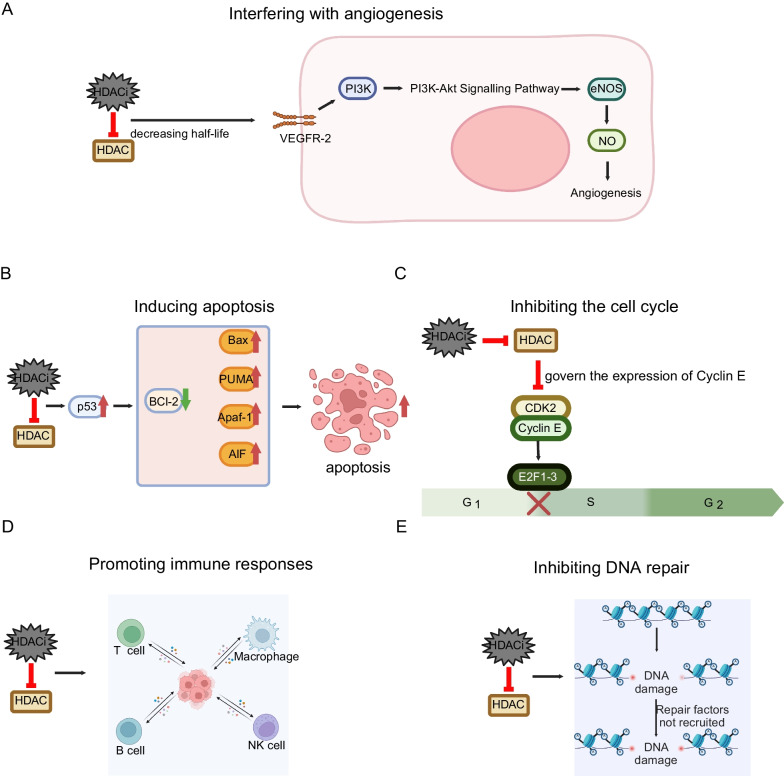
HDACis modulate various biological processes in cancer cells. **A** HDACi treatment reduces angiogenesis by shortening the half-life of VEGFR-2. **B** HDACi-mediated hyperacetylation of key p53 residues enhances the expression of pro-apoptotic genes, thereby promoting apoptosis. **C** HDACi treatment blocks cell cycle progression from G1 to S phase by governing the expression of Cyclin E. **D** HDACi treatment regulates various immune cells to improve immune response. **E** HDACi treatment inhibits DNA repair, leading to persistent DNA damage. Created with BioRender.com

Based on the selectivity of HDACis, HDACis can be categorized into three types: isoform-selective HDACis, class-selective HDACis, and pan-HDACis (Table 2). For instance, Tucidinostat, Entinostat, and Mocetinostat are examples of class-selective HDACis, while Romidepsin, Vorinostat, Panobinostat, and Belinostat are examples of pan-HDACis. Isoform-selective HDACis include TYA-018 and Santacruzamate A, etc. These HDACis work by binding to key Zn^2+^ ions, thereby disrupting the catalytic function of HDAC enzymes and reversing histone deacetylation. This action results in key antitumor effects, such as interfering with angiogenesis, inhibiting the cell cycle, promoting immune responses, inhibiting DNA repair, and inducing apoptosis (Fig. 2).

**Table 2:** The various classes of HDAC inhibitors and their respective stages of development

Drug selectivity	Drug name	Other names	Stage of development	Approval organization	Cancer types
Pan-HDACis	Vorinostat	SAHA, MK0683	Approved	FDA^*^	Cutaneous T-cell lymphoma lymphoma
	Romidepsin	FK228; depsipeptide	Approved	FDA	Cutaneous T-cell lymphoma
	Belinostat	PXD101	Approved	FDA	Adult patients with relapsed or refractory peripheral T-cell lymphoma patients with relapsed or refractory peripheral T-cell lymphoma
	Panobinostat	LBH589	Approved	FDA	Multiple myeloma myeloma
Class-selective HDACis	Tucidinostat	Chidamide; HBI-8000; CS 055	Approved	NMPA^*^	Peripheral T cell lymphoma
	Entinostat	MS-275; SNDX-275	Phase III	/	/
	Mocetinostat	MGCD0103; MG0103	Phase II	/	/
Isoform-selective HDACis	Citarinostat	/	Phase I	/	/

^*^FDA, U.S. Food and Drug Administration; *NMPA, National Medical Products Administration

As of 2009, pan-HDACis constitute the most frequently reported category within the HDACi landscape, while current research is increasingly focused on identifying selective HDAC isoenzyme inhibitors, as they offer improved therapeutic benefits and fewer side effects compared to broad-spectrum HDACis for disease management [43, 44].

**Fig. 3:**
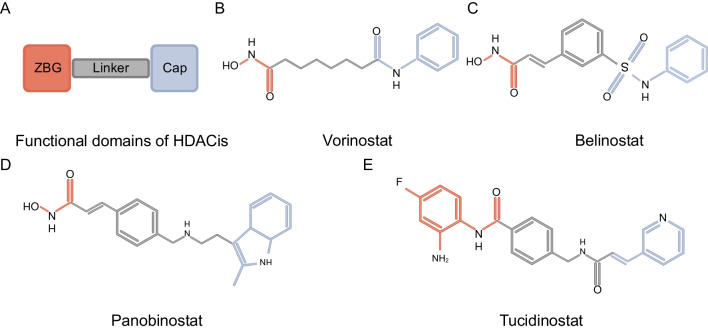
Functional domains of HDACis. HDACis consist of a cap group, a linker, and a ZBG. While the cap and linker influence selectivity and cellular entry, the ZBG is essential for its interaction with the enzyme's zinc ion, crucial for inhibition. Notably, hydroxamates, commonly used as ZBGs, have faced criticism for non-specificity and toxicity. Recent advancements have led to alternative ZBGs like benzamide-based Tucidinostat, maintaining efficacy while mitigating these issues. **A** Pharmacodynamic structure. **B** Vorinostat. **C** Belinostat. **D** Panobinostat. **E** Tucidinostat. Created with BioRender.com

Current HDACis have been developed mostly for metal-dependent HDAC isoenzymes. HDACis are small molecule compounds, and the pharmacodynamic structure of HDACis consists of 3 parts: zinc binding group (ZBG) with the effect of chelating zinc ions at the bottom of the HDAC pocket, a group that acts on the surface recognition area (capping group) at the entrance edge of the HDAC active pocket, and a group that acts on the hydrophobic channel of the active site and connects the link region of the capping group and ZBG [45]. Numerous inhibitors of zinc-dependent HDACs incorporate hydroxamate groups as ZBG [46] (Fig. 3). However, hydroxamates have been found to exhibit a proclivity for nonspecificity, and suspicions are widely held due to their undesirable toxicological effects [46]. Consequently, in recent years, several alternative ZBGs have been synthesized that can substitute for the critical hydroxamate group in HDACis while maintaining high potency. An example of this is the benzamide class HDACi, Tucidinostat. With the advent of new HDACis and the application of enhanced combination therapy strategies, both preclinical research and clinical trials have progressively yielded improved outcomes.

## Preclinical evidence of HDAC inhibitors

We have offered a comprehensive overview of the fundamental mechanism of HDACis. Next, we will integrate preclinical evidence to elucidate the limitations of its efficacy in monotherapy, as well as the enhanced antitumor effects when combining HDACis with other drugs.

### Efficacy of HDACis in monotherapy

As a class of antitumor drugs that have achieved certain efficacy in hematologic malignancies, HDACis as monotherapy for solid tumors have proven to be less effective compared to their use in hematological malignancies [47]. Given that it is well-known that HDACis as monotherapy have not achieved satisfactory results in preclinical studies for the treatment of solid tumors, we devote more space in this section to present the few promising or indicative results from preclinical studies of HDACis as monotherapy in solid tumors. These results, while not directly significant for clinical treatment, provide valuable insights into the pharmacological mechanisms of HDACis' anticancer effects and inform further exploration of combination treatment strategies. For instance, in hepatocellular carcinoma (HCC) cells, Romidepsin has been found to induce apoptosis and G2/M phase arrest through the activation of JNK/c-Jun/caspase-3 and Erk/cdc25C/cdc2/cyclinB pathways, respectively [48]. In various tumors, overexpression of different types of HDACs can occur, necessitating clinical scientists to precisely identify the biological functions of these HDACs. This also places demand on the selectivity of HDACis. For example, a preclinical study focusing on urothelial carcinoma assessed the efficacy of various HDACis. It revealed that the anti-neoplastic effects of compound 19i (LMK235) on urothelial carcinoma cells primarily target class I HDACs. Furthermore, evidence shows that overexpression of HDAC4 does not typically enhance urothelial carcinoma cell proliferation, suggesting that targeting HDAC4 with HDACis may not be effective in treating urothelial carcinoma [49]. In addition to common tumor types, we have also discovered information about molecular subtypes and mutation profiles in the treatment with HDACis. An example of HDACi therapy related to mutation profiles involves Belinostat in KRAS-mutant lung cancer. Belinostat affects cancer metabolism by regulating the Tricarboxylic Acid Cycle, urea cycle, glutathione, and amino acids metabolism, and it inhibits NRF2 signaling to achieve its overall anticancer effects [50]. Similarly, a study has suggested that HDACis could function independently to inhibit both epidermal growth factor receptor (EGFR) and HDAC, potentially offering advantages in the therapies of KRAS mutant colorectal cancer [51]. In a case of molecular subtype, studies have shown that HDACis facilitate the ubiquitination and degradation of KLF5 in basal-like breast cancer. This research unveils a new mechanism through which HDACis suppress basal-like breast cancer, highlighting an innovative interaction between KLF5 protein acetylation and ubiquitination [52]. New HDACis have been continuously reported, such as Z31216525, which has demonstrated efficacious inhibition of ovarian cancer cell proliferation both in vivo and in vitro when applied as monotherapeutic agents [53]. Although HDACi monotherapy has not demonstrated a high proportion of positive outcomes in preclinical studies, the observed anticancer potential has led researchers to pursue further preclinical research on the combination of HDACis with other drugs, yielding results more promising than those of HDACi monotherapy.

### Targeting HDACis for sensitization to chemotherapy

**Fig. 4:**
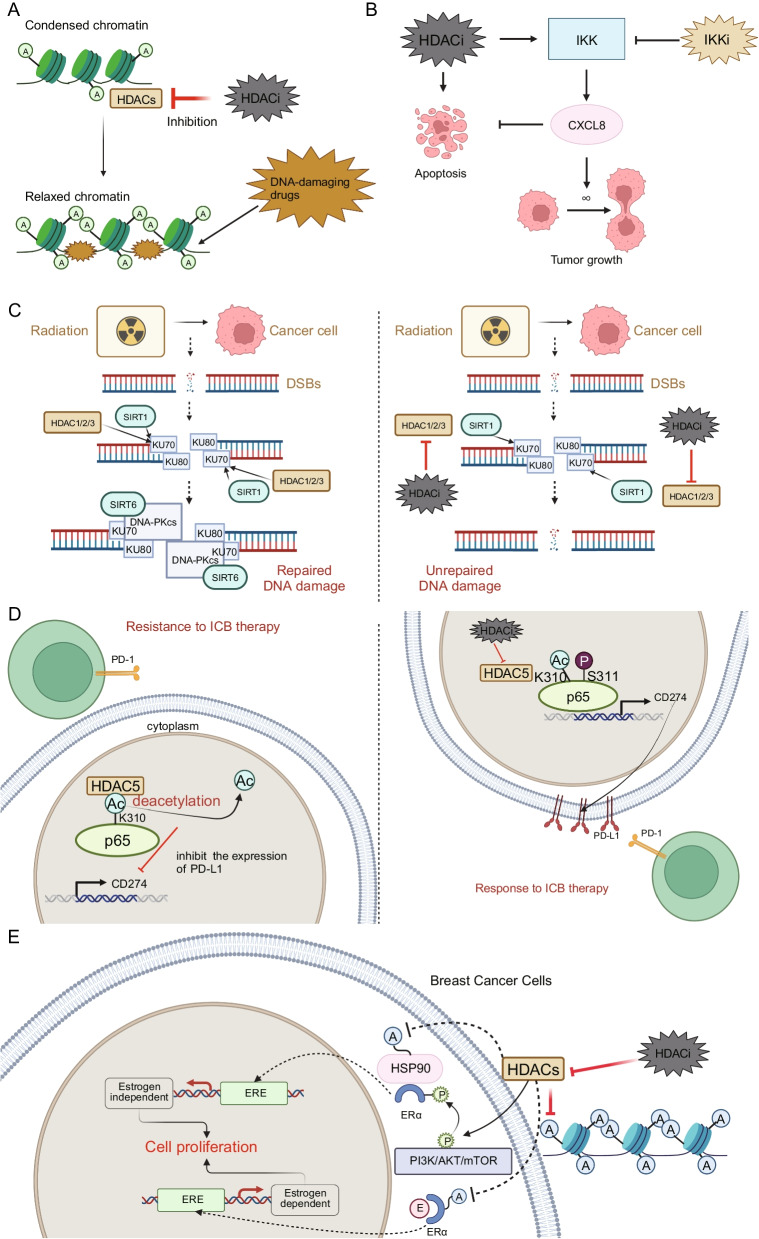
Representative examples of combined therapies involving HDACis in solid tumors. **A** Representative case of HDACi combined chemotherapy: HDACi leads to enhanced DNA damage when mediated by DNA-damaging agents. **B** Representative case of HDACi combined targeted therapy: HDACi in combination with IKK inhibitors promotes apoptosis in cancer cells. **C** Representative case of HDACi combined with radiotherapy: HDACis obstruct the activity of HDAC1, 2, and 3 during the mechanism where HDAC1, 2, 3, and SIRT1 aid in repairing DNA double-strand breaks (DSBs) induced by radiotherapy via nonhomologous end joining. **D** Representative case of HDACi combined immunotherapy: HDACi inhibits HDAC5, and then acetylated p65 is enriched at the gene loci of CD274, promoting the expression of PD-L1 and causing a relatively favorable response to ICB therapy. **E** Representative case of HDACi combined with endocrine therapy: HDACis overcome endocrine resistance by targeting both histone and nonhistone proteins. Created with BioRender.com

In several preclinical studies, HDACis have been described as effectively enhancing the sensitivity to drugs that target DNA. An author mentions in their article that the therapeutic efficacy of HDACis in treating hematologic malignancies and solid tumors may be attributed to their capacity to remodel chromatin, normalize dysregulated gene expression, and inhibit repair of damaged DNA [54]. In addition, the action of HDACis also relaxes the chromatin conformation, making it easier for DNA-damaging drugs to act on the DNA of tumor cells. Guided by such theoretical foundations, HDACis can be effectively combined with various chemotherapy drugs that target DNA. On the one hand, the combined use of HDACis and cisplatin has been shown in studies to sensitize Hela cells to cisplatin therapy [55]. The mechanism by which HDACis sensitize cancer cells to cisplatin has been suggested that HDACis facilitate chromatin relaxation, thereby enhancing the DNA's accessibility to transcription factors. This process may lead to the suppression of specific genes, such as Bcl-2 and XIAP, and support the formation of platinum–DNA adducts [56] (Fig. 4A). The resulting relaxed chromatin and reduced gene expression enhance the efficacy of cytotoxic agents like cisplatin by improving their ability to access DNA and limiting the cellular mechanisms that repair DNA damage [56]. On the other hand, we have taken note of some evidence regarding the combined use of HDACis and doxorubicin, a medication that inhibits the synthesis of RNA and DNA. A study utilizing gene network profiling through the String online network construction tool identified interactions among genes previously associated with resistance to Doxorubicin. The findings suggest that human gastric adenocarcinoma cells can be sensitized to Doxorubicin through concurrent treatment with Cisplatin, an agent that crosslinks within DNA strands, and suberoylanilide hydroxamic acid, a HDACi [57]. Another study focusing on leukemia has revealed that HDACis enhance the sensitivity to Doxorubicin treatment by expanding existing drug-binding sites and establishing new interaction sites, thereby increasing the overall volume of drug-target interactions. This mechanism could also provide valuable insights into sensitization strategies within solid tumors [58]. In conclusion, HDACis can effectively sensitize cells to chemotherapy drugs that act on DNA.

### Targeting HDACis for sensitization to targeted therapy

Due to the complex pathophysiological mechanisms within tumors, cancer cells are prone to developing resistance to single-target drugs, resulting in suboptimal therapeutic outcomes. One approach to improve treatment efficacy is the combined use of multiple targeted therapies [59]. This well-considered approach has presented a rational strategy for enhancing the efficacy of single-target drugs in the context of cancer treatment. HDACis, when used in combination with various other targeted agents such as bromodomain and extraterminal domain (BET) inhibitors, topoisomerase inhibitors, IκB kinase (IKK) inhibitors (Fig. 4B), and receptor tyrosine kinase (RTK) pathway inhibitors, have demonstrated significant antitumor activity in cancer cells from medulloblastoma, glioblastoma, cervical cancer, NSCLC, head and neck squamous cell carcinomas, prostate cancer, HCC, and thyroid cancer [60–63]. Notably, Romidepsin has increased the sensitivity of Erlotinib synergistically in all nine NSCLC cell lines, including EGFR and KRAS wild type cell lines, KRAS mutant cell lines, and TKI resistant EGFR mutant cell lines, with this effect being partially due to enhanced apoptosis [64]. The efficacy of these combination treatments has been well supported by numerous preclinical and clinical studies. Here, we specifically highlight the case of medulloblastoma to demonstrate how combining HDACis can enhance existing targeted therapy approaches. Medulloblastoma is one of the most common malignant brain tumors in children, and despite aggressive current treatments, the prognosis remains poor. Research has shown that HDACis and PI3K antagonists can effectively inhibit the growth of MYC-driven medulloblastoma both in vitro and in vivo, and they suggest that inhibitors of class I HDACs are effective against this type of medulloblastoma [65]. Similarly, a large-scale drug screen has identified selective inhibitors of class I HDACs as a potential therapeutic option for sonic hedgehog medulloblastoma [66]. Additionally, some other relevant research outcomes have even advanced toward further clinical translation. Building upon evidence demonstrating the beneficial impact on cancer treatment outcomes when combining HDACis and RTK pathway inhibitors, clinical translation of these insights is currently being expedited, highlighted by a study that reported the design and synthesis of a novel class of RTK/HDAC dual-targeted inhibitors [61, 67]. Additionally, multitarget drugs containing HDAC inhibition have also been under investigation, such as CUDC-101, which inhibits HDAC, EGFR, and human epidermal growth factor receptor 2 (HER2) [68]. In conclusion, HDACis can synergize with other targeted drugs to obtain stronger antitumor effects.

### Targeting HDACis for sensitization to radiotherapy

The mechanism by which HDACis enhance radiosensitivity is complex and multidimensional. First, following HDAC inhibition, the altered expression or modification of particular genes, including EGFR, AKT, DNA-PK, and RAD51, might impact how cells respond to radiation, resulting in enhanced radiation-induced cytotoxicity in human tumor cells, as has been reported in cell lines of prostate and glioma [69]. Second, several isoforms of HDACs play crucial roles in the mechanisms of DNA repair (Fig. 4C) [34]. This suggests that targeting these particular HDAC isoforms could boost the efficacy of treatments causing DNA damage, encompassing radiotherapy and, as previously mentioned, certain chemotherapy drugs. Additionally, it is intriguing to note that the combination of HDACis with radiotherapy offers some distinct advantages over other single molecularly targeted drugs combined with radiotherapy. The action mechanism of HDACis suggests their potential to enhance radiation response by targeting multiple genes through transcriptional regulation. This encompasses physical modifications to chromatin structure that could alter the susceptibility to radiation damage, as well as differential regulation of oncoproteins after histone acetylation [70]. In summary, the capacity of HDACis to modulate various mechanisms related to radiosensitization renders them highly compatible with the therapeutic objectives of radiotherapy, facilitating their effective integration with such treatments.

### Targeting HDACis for sensitization to immunotherapy

The integration of HDACis with immunotherapy has been substantiated by a body of preclinical evidence. In terms of modulating immune cells, HDAC inhibitors have the capability to enhance the transcription of repetitive elements—short or long patterns of DNA or RNA that occur in multiple copies throughout the genome. This results in the formation of double-stranded RNA and instigates an interferon response. Consequently, this mechanism attracts CD8^+^ T cells and NK cells to combat ovarian cancer in murine models [71]. This highlights the potential of HDACis in enhancing immune-mediated tumor suppression. Furthermore, HDACis induce alterations in gene regulation, leading to an increased expression of genes crucial for facilitating immunorecognition, including natural killer group 2D ligands and heat shock protein70. This consequently amplifies the effectiveness of checkpoint blockade, making bladder cancer cells more susceptible to destruction by T cells [72]. Additionally, there are studies indicating that utilizing IL21 in conjunction with HDACis for in vitro cultivation results in T-cell populations with enhanced persistence, which can potentially boost the performance of adoptively transferred T cells [73]. In the case of macrophages, HDACis render them vulnerable to phagocytosis induced by anti-CD47 through tumor inflammation driven by the NF-kB-TGM2 pathway [74]. Research has also revealed that Vorinostat plays a role in reducing the polarization towards M2 macrophages through the ARID1A^6488delG^/HDAC6/IL-10 signaling pathway in ovarian cancer linked to endometriosis. This presents a novel approach in immunocyte-associated therapeutic strategies [75]. Additionally, HDACis were reported to induce an antitumoral phenotype in macrophages and ameliorate the suppressive tumor microenvironment [76]. One study revealed that incorporating Vorinostat presents a straightforward and potent method to navigate through the critical transition phase from pluripotent stem cells to CD34^+^CD45^+^ hematopoietic stem and progenitor cells, paving the way for readily available cellular immunotherapy [77].

In terms of modulating tumor cells, HDACis influence PD-L1 expression and acetylation-dependent PD-L1 nuclear translocation by targeting and inhibiting HDAC, thereby affecting tumor cell responsiveness to immune checkpoint blockade (ICB) therapies (Fig. 4D) [40, 78, 79]. Regarding the specific mechanism, research indicates that HDAC3 and its associated corepressor SMRT are recruited to the PD-L1 promoter by the transcriptional repressor BCL6. Additionally, inhibition of HDAC3 decreases DNA methyltransferase 1 protein levels, indirectly activating PD-L1 transcription. Finally, this inhibition also boosts PD-L1 expression on dendritic cells within the tumor microenvironment, further supporting the therapeutic potential of targeting HDAC3 in enhancing immune responses [80]. Another mechanism related to cancer cells involves short chain fatty acids acting as HDACis in colorectal cancer cells, which exhibit microsatellite instability and deactivate DNA mismatch repair. This provokes DNA damage, leading to the upregulation of chemokines, MHCIs, and genes associated with antigen processing or presentation, and consequently enhancing immune surveillance mechanisms [81]. In summary, HDACis are highly synergistic with immunotherapeutic approaches by modulating the antitumor activity of various immune cells, enhancing immune surveillance, and inducing tumor cells to express PD-L1, among other methods.

### Targeting HDACis for sensitization to endocrine therapy

As two major classes of drugs in breast cancer treatment, whether HDACis and endocrine drugs can be combined for the treatment of breast cancer is worth exploring. Research has shown that HDAC1 forms a complex with MTA2 and is associated with SETD6, which has been demonstrated to control the expression of estrogen-responsive genes and the proliferation of breast carcinoma cells [82]. Therefore, by specifically inhibiting HDAC, HDACis could remove epigenetic silencing and improve the sensitivity of breast cancer cells to endocrine therapy to a certain extent (Fig. 4E) [83]. In addition, the sensitivity can be re-achieved by HDACis through targeting nonhistone proteins, such as AKT and hypoxia-inducible factor 1-alpha (HIF1α) (Fig. 4E) [84, 85]. In another study, researchers identified a distinctive mechanism underlying Tamoxifen resistance in receptor-positive breast cancer. This resistance is attributed to ZEB1-mediated histone deacetylation and DNA methylation of the MIR497HG promoter. Intriguingly, the application of HDAC1/2 inhibitors was found to boost MIR497HG expression in MCF7/TamR cells, suggesting a potential avenue to combat tamoxifen resistance [86]. Overall, several preclinical research findings have demonstrated that the combination of HDACis and endocrine therapy has exhibited promising antitumor effects in breast cancer, warranting further exploration in clinical research.

## Clinical trials of HDAC inhibitors in solid tumors

While the "Preclinical evidence of HDAC inhibitors" section reports effective anti-tumor activity of HDACis in various solid tumor cases, most studies show positive outcomes, with some exceptions [87]. This highlights the imperfections in the preclinical evidence supporting the use of HDACis in treating solid tumors. Notably, the limitations of preclinical cancer research models, which do not always effectively translate into clinical trials, are evident. These discrepancies may include, but are not limited to: the significantly smaller sample sizes in preclinical studies compared to clinical trials; the standard conditions under which most preclinical experiments are conducted that do not mimic clinical settings; or the differences in tolerance to toxic effects between experimental animals and humans [87].

Given these considerations, conclusions from preclinical studies should be regarded only as references, setting expectations for clinical trial outcomes. As detailed in the subsequent sections, both failures and successes in clinical settings underscore the critical need to thoroughly re-examine the current state of clinical research on HDACis. Therefore, an urgent reassessment is required to understand the full potential and limitations of HDACis in clinical settings. To this end, several different HDACis have already been evaluated in clinical trials, highlighting the ongoing efforts to refine treatment strategies and enhance patient outcomes [88, 89]. As of present, Clinicaltrials.gov has registered hundreds of clinical trials pertaining to HDACis. This review covers several clinical trial examples of using HDACis for the treatment of solid tumors. Additionally, we have included a few trials involving HDACi treatment of hematologic malignancies in a table to highlight that the therapeutic efficacy of HDACis in solid tumors is not inferior to that in hematologic malignant tumors. (Table 3). Furthermore, this section discusses several emblematic clinical research cases to indicate the evolving trends in HDACi applications.

**Table 3:** Representative clinical trials targeting HDACs^*^

Condition or disease	Target	HDACis involved	Phase	Efficacy	References
Breast cancer	HDAC isoenzymes 1, 2, 3 and 10	Tucidinostat	III	Tucidinostat vs. placebo in breast cancer improved PFS (7.4 vs. 3.8 months)	[90]
HR+/HER2-, advanced invasive breast cancer	HDAC isoenzymes 1, 2, 3 and 10	Tucidinostat	II	Tucidinostat combined with fulvestrant showed encouraging antitumor activity and tolerable toxicity in patients with HR + /HER2- advanced breast cancer that had progressed after previous endocrine therapy	[91]
HR+/HER2-, and node-positive, stage II–III breast cancer	HDAC isoenzymes 1, 2, 3 and 10	Tucidinostat	II	The combination of Tucidinostat and exemestane achieved an ORR of 40% and DCR of 100%	[92]
HR+/HER2- and stage II/III breast cancer	HDAC isoenzymes 1, 2, 3 and 10	Tucidinostat	II	The combination of Tucidinostat and exemestane achieved an ORR of 75% (18/24). The DCR was 100%	[93]
Unresectable or advanced melanoma	HDAC isoenzymes 1, 2, 3 and 10	Tucidinostat	I b/II	Tucidinostat in combination with nivolumab is well tolerated and demonstrates very encouraging efficacy in patients with anti-PD1-naïve advanced melanoma	[94]
Unresectable or metastatic melanoma	class I HDAC (HDAC1, HDAC2 and HDAC3)	Entinostat	–	Entinostat in combination with PEMBRO demonstrates significant clinical activity and acceptable safety (The median PFS is 4.2)	[95]
HR+/HER2- breast cancer	class I HDAC (HDAC1, HDAC2 and HDAC3)	Entinostat	III	Entinostat vs. placebo in breast cancer improved PFS (6.32 vs. 3.72 months)	[96]
Urothelial carcinoma	HDAC isoenzymes 1, 2, 3 and 11	Mocetinostat	II	The efficacy observed was considered insufficient to warrant further investigation of mocetinostat as a single agent in this setting	[8]
Peripheral T-cell lymphoma	HDAC isoenzymes 1, 2, 4 and 6	Romidepsin	II	PFS was 4.6 months in the older population and 3.7 months in the younger population The median OS was 11.8 months in both the older and younger populations	[97]
High-risk lymphoma	Pan HDAC	Vorinostat	I	The observed toxicities make Vorinostat unsuitable for long-term maintenance therapy	[98]
Children With Relapsed Solid Tumor, Lymphoma or Leukemia	Pan HDAC	Vorinostat	I and II	Best overall response (combining PR and SD, no CR observed) rate in phase II was 6/27 (22%) with a median PFS and OS of 5.3 and 22.4 months	[99]
Advanced thymic epithelial malignancies	Pan HDAC	Belinostat	II	Belinostat has modest antitumor activity in this group of heavily pretreated thymic malignancies (patients with thymoma has a PFS of 5.8 months) (patients with thymic carcinoma has a PFS of 19.1 months)	[100]
Relapsed or refractory peripheral or cutaneous T-cell lymphoma	Pan HDAC	Belinostat	II	ORRs were 25% (PTCL) and 14% (CTCL)	[101]
Refractory metastatic renal cell carcinoma	Pan HDAC	Panobinostat	II	Panobinostat had no activity in this group of patients with refractory renal carcinoma	[102]
Previously treated advanced solid tumors	HDAC6	Citarinostat	Ib	The combination of citarinostat plus paclitaxel showed an acceptable safety profile	[103]
Metastatic urothelial, renal and prostate carcinoma	Pan HDAC	Vorinostat	Ib	The combination of vorinostat and pembrolizumab is relatively well tolerated and may be active in a subset of immune checkpoint resistant UC/RCC patients and immune checkpoint naïve PCA patients	[104]
Patients with melanoma who have progressed on prior PD-(L)1 blockade	class I HDAC (HDAC1, HDAC2 and HDAC3)	Entinostat	Ib/ II	Entinostat + Pembrolizumab continues to demonstrate promising anti-tumor activity and acceptable safety in patients with melanoma who have progressed on prior PD-(L)1 blockade	[105]
Children and Adults with Recurrent Medulloblastoma	Pan HDAC	Panobinostat	I	–	NCT04315064
MSS/pMMR colorectal cancer	HDAC isoenzymes 1, 2, 3 and 10	Tucidinostat	II	The combination of a PD-1 antibody, an HDACi, and a VEGF antibody could be a promising treatment regimen for patients with MSS/pMMR advanced colorectal cancer	[106]
Advanced Epithelial Ovarian Cancer	class I HDAC (HDAC1, HDAC2 and HDAC3)	Entinostat	I and II	The Avelumab + Entinostat group exhibited improved ORR and PFS	NCT02915523

^*^Clinical trials with results reported were summarized. Representative trials were selected for illustration due to the limited space

*HDAC* Histone deacetylase; *PFS* Progression-free survival; *HR* Hormone receptor; *HER2* Human epidermal growth factor receptor 2; *ORR* Objective response rate; *DCR* Disease control rate; *PD1* Programmed cell death protein 1; *PR* Partial response; *SD* Stable disease; *CR* Complete response; *PTCL* Peripheral T-cell lymphoma; *CTCL* Cutaneous T-cell lymphoma

### Clinical trials of selective HDACis

As selective HDACis have experienced rapid advancement, clinical trials for selective HDACis have gradually commenced, these selective inhibitors have also shown promising prospects in clinical studies related to solid tumors.

Promising clinical trial results have already been achieved in many cancers, particularly in HR-positive, HER2-negative (HR+/HER2-) breast cancer, which stands out as a notable example. As a commonly used endocrine medicine in postmenopausal women with HR+/HER2- breast cancer, Exemestane has established a significant position in clinical practice for these patients. Consequently, researchers have initiated several clinical trials to evaluate the efficacy and safety of combining HDACis with Exemestane in the treatment of HR+/HER2- breast cancer. As a result, in the sphere of selective HDACis, particularly Tucidinostat and Entinostat, we have observed a promising trend in the treatment of HR+/HER2- breast cancer. A single-center, prospective, open-labeled, single-arm phase II study (ChiCTR2100046678) was conducted on patients with HR+/HER2-, and node-positive, stage II–III breast cancer. The objective response rate (ORR) was 40%, with a Complete Remission (CR) of 5% (n = 1) and a Partial Response (PR) of 35% (n = 7). The disease control rate (DCR) was 100% [92]. Another trial, the open-label, single-center, phase II NeoTEE study, similarly showed the efficacy of Tucidinostat plus Exemestane in treating patients with HR+/HER2-, stage II/III breast cancer [93]. 18 out of 24 patients achieved a PR, resulting in an ORR of 75% (18/24). The DCR was 100%. The above two early-phase trials involving Tucidinostat and Exemestane reported impressive DCRs of 100% in both studies. However, a stark contrast was observed in their ORRs: one trial reported an ORR of 40%, while the other demonstrated a significantly higher ORR of 75%. The large difference in ORR between two similarly designed trials could be attributed to a difference in the enrolled patients. The ChiCTR2100046678 trial required node-positive patients, whereas NeoTEE did not. Nevertheless, the researchers of both clinical trials consider the combination of Tucidinostat and Exemestane to have good tolerability and to demonstrate an encouraging clinical response in HR+/HER2- breast cancer. Encouraged by multiple positive clinical outcomes, a large-scale, randomized, double-blind, placebo-controlled, phase III ACE trial was initiated to confirm the efficacy of Tucidinostat plus Exemestane in patients with HR+/HER2- breast cancer, targeting postmenopausal women with HR+/HER2- breast cancer [90]. The trial enrolling 365 patients further corroborated the efficacy of Tucidinostat combined with Exemestane. The Tucidinostat treatment showed an investigator-assessed PFS of 7.4 months, compared to 3.8 months in the placebo group. These results suggest that combining Tucidinostat with Exemestane can effectively improve PFS in postmenopausal patients with advanced HR+/HER2- breast cancer. In addition to Tucidinostat, another selective HDACi, Entinostat is also being explored in clinical trials to treat HR+/HER2- breast cancer. In a randomized, double-blind, placebo-controlled Phase III trial conducted across 35 sites in China with 354 patients, it was found that combining Entinostat with Exemestane significantly improved PFS in advanced HR+/HER2- breast cancer patients who had relapsed or progressed after endocrine therapy. The median PFS was 6.32 months (95% CI 5.30–9.11) in the Entinostat group, compared to 3.72 months (95% CI 1.91–5.49) in the placebo group [96]. Fortunately, it is evident that both Tucidinostat and Entinostat have demonstrated varying degrees of benefit for HR+/HER2- breast cancer patients, making them worthy of consideration for clinical application. Moreover, based on a comparison of some clinical trial evidence, HDACis have demonstrated a certain degree of superior efficacy in treating HR+/HER2- breast cancer compared to some other therapies. Take an example of another scheme of therapy, the combination of steroidal Aromatase Inhibitors (AI) and Tucidinostat was given a Class I recommendation for postmenopausal HR + patients who had an inadequate response to nonsteroidal AI treatments. In another trial, the ELAINE 2 phase II study, 29 women with HR+/HER2- ESR1-mutated metastatic breast cancer were treated with a combination of Lasofoxifene and Abemaciclib [107]. This treatment approach results in a median PFS of 13.9 months and an ORR of 33.3%. By comparison, the combination of HDACis with Exemestane in treating HR+/HER2- breast cancer can achieve an impressive ORR of up to 75%, establishing it as a potentially better choice for treatment in certain cases of HR+/HER2- breast cancer. Supported by these encouraging clinical trial results, the breast cancer treatment guidelines by the Chinese Society of Clinical Oncology designated the combination of AI and Tucidinostat as a Class I recommendation for postmenopausal patients with HR + who did not respond to tamoxifen treatment. Besides breast cancer, clinical trials involving selective HDACis are also being conducted for other tumor types. For instance, in the randomized phase 2 CAPability-01 trial, researchers assessed the effectiveness of combining the PD-1 monoclonal antibody Sintilimab with the HDACi Tucidinostat, with or without the anti-VEGF monoclonal antibody Bevacizumab, in patients with unresectable chemotherapy-refractory locally advanced or metastatic microsatellite stable/proficient mismatch repair (MSS/pMMR) colorectal cancer. The findings suggest that this combination of a PD-1 antibody, an HDACi, and a VEGF antibody could represent a promising treatment approach for patients with advanced MSS/pMMR colorectal cancer [106]. Additionally, a randomized, placebo-controlled, double-blind, multicenter Phase 1b/2 study, registered under NCT02915523, investigated Avelumab with or without Entinostat in patients with advanced epithelial ovarian cancer. The study reported a median PFS of 1.64 months for the Avelumab + Entinostat group, compared to 1.51 months for the Avelumab + Placebo group. Furthermore, under the criteria of RECIST 1.1 for assessing the ORR, the ORR for the Avelumab + Entinostat group was 5.88%, while it was 4.88% for the Avelumab + Placebo group. Additionally, a clinical study revealed the efficacy and safety of Entinostat and Pembrolizumab in patients with melanoma who are progressing on or after treatment with a PD-1/L1 blocking antibody. The results indicate that Entinostat combined with Pembrolizumab continues to exhibit promising anti-tumor activity and acceptable safety in patients with melanoma who have progressed on previous PD-L1 blockade therapy [105]. As HDACis evolve with enhanced and refined selectivity, such specificity may pave the way for their future development and utilization, potentially resulting in fewer side effects and more targeted treatments. While current online resources have offered limited clinical trial results on single-isoform selective HDACis, a few promising and representative clinical studies hint at a bright future for these inhibitors [103].

Of course, not all clinical trials related to selective HDACis have yielded satisfactory results. The following are some representative clinical trials that did not achieve favorable outcomes. A trial on Mocetinostat demonstrates its inadequacies as a monotherapy. In a trial of patients with previously treated, locally advanced/metastatic urothelial carcinoma with inactivating alterations in acetyltransferase genes, the low responsiveness of Mocetinostat as a monotherapy was found insufficient for further investigation. This led to the cancellation of an anticipated phase II trial, as they believed that the observed therapeutic effects did not warrant pursuing Mocetinostat as a monotherapy in this setting [8]. This scenario indicated that even selective HDACis, when used as standalone treatments, do not always guarantee success. While combining other therapies with selective HDACis could be a potential strategy to address treatment challenges, it may also not always yield satisfactory results in treating solid tumors. Another research study is conducting a Phase 1b/2 randomized, placebo-controlled, double-blind, multicenter trial evaluating the combination of the immunotherapy agent Atezolizumab with or without Entinostat in patients with advanced triple-negative breast cancer [108]. Results from this trial revealed that patients receiving the combination of Entinostat and Atezolizumab experienced a PFS of 1.68 months, compared to 1.51 months in the placebo group. However, these results did not reach statistical significance in terms of the p-value. This trial revealed that in patients with previously treated advanced TNBC, the addition of Entinostat to Atezolizumab did not extend the median PFS compared to Atezolizumab and placebo, and the combination resulted in increased toxicity. This may indicate that Entinostat is insufficient in sensitizing the effects of immunotherapy when used in combination, failing to achieve a synergistic effect in treatment. Personalized combination treatments involving Entinostat should be considered to optimize its effectiveness. If the aforementioned cases primarily showcase the limitations of the therapeutic efficacy of many HDACis, then the following trial highlights another weakness of HDACis: their toxic side effects. In this trial, the failure of the randomized phase I study of Regorafenib, Hydroxychloroquine (HCQ), and Entinostat in metastatic colorectal cancer has raised concerns about the development of selective HDACis [109]. This experiment was based on a series of preliminary studies on drug tolerance, specifically showing that the addition of HCQ to various cancer regimens resulted in relatively mild toxicity. The HDACi Vorinostat combined with HCQ was also well-tolerated in colorectal cancer. It can be considered that the individual drugs used in combination therapy before this clinical trial did not exhibit unacceptable toxicity. However, when these three drugs were used together in treatment, unexpected, intense, and irreversible side effects led to the failure of the clinical trial. Upon further investigation, it was found that the evidence relied upon for assessing the feasibility of combining Regorafenib and Entinostat was based solely on a tyrosine kinase inhibitor (TKI) similar to Regorafenib, Sorafenib. The combination of Entinostat and Sorafenib, administered every two weeks, did not show toxicity exceeding the expected levels for each drug at full dosage. It is possible that subtle differences between Regorafenib and Sorafenib may lead to various pharmacokinetic and pharmacodynamic effects, ultimately resulting in unpredictable toxicity. The occurrence of this unexpected situation serves as a reminder of the importance of considering pharmacological properties, such as drug interactions, when administering combination therapies. These interactions include those based on pharmacodynamics and pharmacokinetics, as well as direct interactions between drugs. Notably, the risk of a drug-drug interaction increases with the number of drugs used [110]. Therefore, it is crucial to conduct further research to thoroughly understand the toxicity of combination therapies. This will aid in the selection of more effective drug regimens and enhance their practicality. Conducting repeated preclinical studies in various settings will also help to minimize potential risks in clinical practice.

In general, selective HDACis have demonstrated promising antitumor efficacy in clinical trials pertaining to an array of solid tumors, especially in combination therapy modalities. The selectivity of selective HDACis offers a unique advantage for precision oncology over pan-HDACis. However, the clinical application of selective HDACis also demands extensive characterization of each HDAC isoform's physiological functions and a comprehensive understanding of the HDAC expression landscape across various cancers. Overall, using selective HDACis for precision cancer treatment necessitates a customized approach tailored to individual patient conditions, representing a promising future direction for therapy.

### Clinical trials of pan-HDACis

Indeed, a significant portion of research has focused on the application of pan-HDACis, which make up the bulk of HDACis presently accessible in clinical environments [111, 112].

Among the numerous completed clinical trials, those involving Vorinostat constitute a significant proportion. Therefore, we will focus our discussion on Vorinostat, a highly representative pan-HDAC inhibitor. Vorinostat is an oral pan-HDACi that targets a wide range of HDACs. Vorinostat inhibits HDACs to induce hyperacetylation of histones and many other cytoplasmic proteins, leading to cell cycle arrest, apoptosis, autophagy, and cell death. A phase I clinical trial in the treatment of gastrointestinal cancer suggested Vorinostat as a potentially active agent in the treatment of gastrointestinal cancer [113]. Additionally, clinical trials have proven that the combination of Vorinostat and I-metaiodobenzylguanidine is more effective in treating neuroblastoma than its standard therapy. In a phase II clinical trial for patients with relapsed or refractory neuroblastoma, the combination of Vorinostat and I-metaiodobenzylguanidine demonstrated a higher true response rate than the combinations of Vincristine with I-metaiodobenzylguanidine or Irinotecan with I-metaiodobenzylguanidine [114]. Clinical research on Vorinostat, including its application in urogenital cancers such as prostate cancer, has been conducted. A phase Ib study involving Vorinostat and Pembrolizumab in patients with metastatic urothelial, renal, and prostate carcinoma suggests that the combination is generally well tolerated and shows potential efficacy in a subset of patients resistant to immune checkpoint inhibitors with urothelial and renal carcinomas, as well as in those naive to such treatments with prostate carcinoma [104]. Another phase II clinical trial, reported on the combination of Pembrolizumab and Vorinostat in recurrent/metastatic squamous cell carcinomas of the head and neck (HNSCC) also demonstrated the superiority of HDACi treatment in solid tumors [115]. In this clinical trial, patients with HNSCC treated with Pembrolizumab plus Vorinostat showed an ORR of 38.1% (8 out of 21 patients). By comparison, previous reports indicated that response rates to Pembrolizumab as a monotherapy were 23%, significantly lower than 38.1% [116]. Even the 36% rate for standard treatment is less than 38.1% [116]. This finding suggested that Vorinostat can significantly enhance the therapeutic effectiveness of Pembrolizumab in treating HNSCC, although toxicities were higher than those reported with Pembrolizumab alone. In our previously discussed context of medulloblastoma, several clinical trials are currently underway. For this type of tumor, which current treatments cannot effectively cure and which causes significant suffering to patients, the development of new and effective treatment methods is particularly meaningful. For instance, trial NCT04315064, which includes children and adults with recurrent medulloblastoma, is exploring treatment with Panobinostat. Should this trial achieve notable success, it could potentially contribute to better standardized treatment protocols and offer new hope for managing this disease.

However, pan-HDACis have also faced many setbacks in clinical trials for the treatment of solid tumors. The failure of the randomized phase III, double-blind, placebo-controlled trial of Vorinostat in patients with advanced malignant pleural mesothelioma who had progressed on previous chemotherapy (VANTAGE-014) cast a shadow on the development of pan-HDACis [117]. Several factors contributed to the failure of the VANTAGE-014 trial. Firstly, the trial lacked robust biomarkers to accurately identify patients who could benefit from Vorinostat. The design concept of the VANTAGE-014 trial was based on an original phase I trial of the oral formulation, in which Vorinostat showed preliminary signs of clinical activity in patients with malignant pleural mesothelioma [118]. However, this original trial was a small-scale study involving only 73 patients and did not mention being multi-centered. Consequently, the favorable results observed might not be exclusively attributed to the incorporation of Vorinostat. Instead, they could have been influenced by the presence of an especially advantageous patient group, which could occur randomly or due to unregulated positive selection in the recruitment process. Secondly, the adverse outcome of the VANTAGE-014 trial might also be attributed to insufficient exposure to Vorinostat, following the approved dosing schedule for cutaneous T-cell lymphoma. However, it remains unclear whether malignant pleural mesothelioma and cutaneous T-cell lymphoma share the same sensitivity to Vorinostat, hence this dosing schedule might not be adequate for inducing sustained targeted HDAC inhibition. Additionally, the lack of personalized combination therapy strategies might have led to reduced efficacy of Vorinostat. Preclinical studies suggest that various HDACis, including Vorinostat, in combination with chemotherapy, targeted therapy, radiotherapy, and immunotherapy, show promising results. Therefore, personalized combination treatments with Vorinostat should be considered to optimize its effectiveness. Furthermore, additional phase II clinical trials have yielded results indicating that Vorinostat in the treatment of solid tumors may or may not improve prognosis. For instance, the combination regimen with Bevacizumab and Vorinostat is well tolerated in patients with recurrent glioblastoma. However, this combination therapy for this study population did not improve PFS or OS when compared with Bevacizumab monotherapy [119].

In summary, while pan-HDACis have demonstrated promising results in certain solid tumors, they have not achieved satisfactory anticancer effects in some instances.

### Discussion on the trials

In summary, an in-depth analysis of clinical trials involving HDACis for solid tumors reveals a complex and nuanced landscape of cancer treatment. However, these findings also bring to light the critical necessity for personalized treatment strategies. Among the numerous categories of solid tumors known to humans, HDACis have shown good efficacy in some solid tumors in clinical or preclinical settings, but have not demonstrated consistently positive results in others. This limitation may be due to factors such as varying levels of HDAC expression or because the maximum tolerable doses fall below the levels required for effective anti-tumor activity in some types of solid tumors. This situation encourages drug researchers to develop better, less toxic HDACis, prompts clinical researchers to design more effective treatment regimens, and urges basic medical researchers to more deeply investigate the various properties of HDACs and HDACis. Driven by this vision, the development of HDACis is increasingly focusing on selective inhibitors, which target more precisely, attracting growing research interest. Selective inhibitors, such as Tucidinostat and Entinostat, have demonstrated notable efficacy in specific types of cancer. However, their effectiveness is not uniformly applicable across all solid tumor types. On the other hand, pan-HDACis, characterized by their broader spectrum of action, offer a different array of therapeutic benefits and challenges. This comprehensive analysis of HDACis in the context of solid tumors not only emphasizes the importance of tailoring treatment to the specificities of each cancer type but also lays the groundwork for future research. Such research is essential for optimizing the use of HDACis in personalized cancer therapy, aiming to maximize efficacy while minimizing adverse effects.

The deepening insight into these inhibitors within clinical settings illuminates the ever-evolving landscape of cancer treatment, underscoring the continuous pursuit of tailoring the most effective therapeutic approach to each individual patient.

## Factors limiting the clinical application of HDAC inhibitors

While HDACis have shown anticancer effects against certain types of solid tumors in some preclinical and clinical studies, there are still significant factors that constrain their application and widespread adoption. These include toxic side effects, tumor heterogeneity, and off-target effects.

First, toxic side effects limit the clinical application of HDACis. Researchers have noted that while toxicities associated with HDACis in clinical settings are largely manageable, there are cases where cardiotoxicities, as well as constitutional, hematologic, and gastrointestinal effects, may become dose-limiting. This highlights the nuanced management of dosages in clinical applications of HDACis. This also underscores the importance of further evaluating the toxic side effects of HDACis to understand their impact on clinical applications [120]. A previously mentioned clinical study reported common adverse events observed in both the combination arms—Sintilimab and Tucidinostat (23 patients), and Sintilimab, Tucidinostat, and Bevacizumab (25 patients). These included proteinuria, thrombocytopenia, neutropenia, anemia, leukopenia, and diarrhea. Notably, the study recorded two treatment-related fatalities, one due to hepatic failure and the other to pneumonitis. While the authors suggest that the regimen combining a PD-1 antibody, an HDACi, and a VEGF antibody holds promise for treating patients with MSS/pMMR advanced colorectal cancer, the fatalities underline the potential risks associated with this combination therapy in treating colorectal cancer [106]. Furthermore, the teratogenic potential of HDACis has been documented. Compounds such as valproic acid, trichostatin A, apicidin, MS-275, sodium butyrate, boric acid, and salicylic acid have been implicated in causing congenital malformations through histone hyperacetylation in target organs. A major consequence observed a few hours after embryonic exposure to these HDACis is cell death in the target organs. The hypothesized mechanisms of action leading to HDACi-induced teratogenic effects include gene deregulation, oxidative stress, DNA demethylation, and retinoic acid imbalance [121]. Moreover, a meta-analysis incorporating four studies with a total of 1,457 patients revealed that the HDACi plus endocrine therapy (HE) regimen resulted in increased toxicity compared to the placebo plus endocrine therapy (PE) group. This was evident in higher rates of overall adverse events, Grade ≥ 3 adverse events, dose modifications, and discontinuation rates [122]. Many researchers have underscored the importance of predictive biomarkers of HDACi toxicity to facilitate personalized treatment strategies. Overall, while HDACis hold significant potential in cancer therapy, careful monitoring and management of toxicities are crucial, and identifying predictive biomarkers can optimize their therapeutic benefits. One review has pointed out that a second generation of orally available HDACis has been developed, drawing from the chemical structures of clinically effective agents like hydroxamic acids and benzamides. Although research is still ongoing, early preclinical studies suggest these new agents are more potent than their predecessors, offer better pharmacodynamic and pharmacokinetic profiles, and are potentially less toxic [120]. This may also suggest that the development of HDACis is progressing towards reducing adverse events to achieve wider clinical use.

Second, phenotypic heterogeneity in tumor cells leads to therapeutic resistance [123]. Alterations in HDAC expression have been commonly observed in both hematologic malignancies and solid tumors [124, 125]. As a class of antineoplastic agents targeting HDACs, HDACis may experience variable therapeutic efficacy due to differential expression levels of HDACs across diverse tumor types. Such heterogeneity of tumor cells could attenuate the therapeutic impact of HDACis, thus constraining their applicability in malignancies characterized by low HDAC expression. Moreover, the inherent heterogeneity of tumor cells might facilitate the occurrence of relapses and confer resistance to single-target drugs [59]. In scenarios where the tumor has no other therapeutic targets available, combination targeted therapies involving HDACis are difficult to carry out. Additionally, the synergistic potential of HDACis with immunotherapy is rooted in their dual action on both immune and tumor cells. Moreover, certain tumors exhibit immune-desert profiles, characterized by mechanisms that evade immune recognition and suppress activation of the immune system [126]. In such contexts, the regulatory influence of HDACis on immune cells becomes markedly constrained, thereby limiting their therapeutic efficacy. These complexities have highlighted the multifaceted challenges posed by tumor heterogeneity in optimizing HDACi therapy.

Third, off-target effects limit the clinical application of HDACis. It has been reported that in breast cancer models, HDAC inhibition elevates histone acetylation at the LIFR gene promoter and triggers the JAK1-STAT3 signaling pathway [127]. This suggested that HDACis might also lead to the triggering of other procancer signaling pathways. Such circumstances have prompted researchers to consider combined treatments using HDACis and methods such as JAK1 or BRD4 inhibition to counteract resistance to HDACis. The off-target effects of HDACis have been a significant factor currently limiting their application. Combining them with other targeted agents might offer a potential avenue to overcome this limitation to some extent.

Overall, the widespread application of HDACis still faces numerous challenges and limitations. Strategies such as combination therapies and the development of more targeted HDACis are needed to overcome these challenges.

## Future directions: development trends in HDAC inhibitors

In the realm of cancer therapy, the utilization of HDACis in solid tumors is garnering increasing attention. Despite the fact that preclinical studies of HDACis monotherapy for treating solid tumors have not achieved the same level of success as those for hematologic malignancies, and clinical trials in solid tumors have shown mixed results and limited success, the efforts are nonetheless of significant value [128]. The extensive research and exploration conducted by scientists continue to provide valuable insights into the ongoing development of HDACis. The future outlook for HDACis can be concisely encapsulated in three key areas: the enhancement of combination therapy approaches, the innovation of new drug formulations and the identification of more precise biomarkers. An increasing number of new combinatorial approaches involving HDACis and other antitumor agents are under investigation to leverage synergistic effects and potentially surmount drug resistance. Additionally, novel strategies for HDACi drug delivery have been explored, such as antibody drug conjugate delivery systems, which show promise in optimizing the pharmacokinetics and bioavailability of HDACis, thereby directing drug delivery to tumor sites [129]. Furthermore, researchers have been exploring and innovating novel HDACi drug types to address prevailing challenges and amplify therapeutic efficacy [130]. A key area of interest was the development of class-specific or isoform-selective HDACis, which aim to increase target specificity while minimizing off-target effects [131]. The integration of these forward-looking initiatives is poised to significantly advance HDACi drug development and ultimately improve clinical outcomes for cancer patients.

## Conclusions

In the current medical landscape, HDACis have emerged as a promising therapeutic option for the treatment of select solid tumors, a development substantiated by a series of encouraging results from preclinical studies and considerable clinical trial evidence. Concurrently, there is a robust pipeline of novel HDACis under active development, and an increasing number of sophisticated combinational treatment strategies involving HDACis are being explored and optimized. These trends collectively signify the promising future applications of HDACis in oncology. However, several challenges need to be addressed for the optimal utilization of HDACis, including toxic side effects, tumor heterogeneity, and potential off-target effects. We believe that as research progresses, these challenges will be partially overcome, and the ongoing exploration of HDACis is expected to have a positive impact on the future clinical treatment of an increasing number of solid tumors.

## Abbreviations

IKK: I kappa B kinase
CXCL8: C-X-C Motif Chemokine Ligand 8
DSBs: DNA double-strand breaks
SIRT: Sirtuin
DNA-PKcs: DNA-dependent protein kinase catalytic subunit
ICB: Immune checkpoint blockade
ERE: Estrogen response element
Erα: Estrogen receptor α
e: Estrogen
BAX: Bcl-2-associated X protein
AIF: Apoptosis inducing factor
BCL2: B-cell lymphoma 2
APAF: Apoptotic protease activating factor-1
PUMA: P53 upregulated modulator of apoptosis
eNOS: Endothelial nitric oxide synthase
PI3K: Phosphoinositide 3-kinases
Akt: RAC-alpha serine/threonine-protein kinase
CDK: Cyclin-dependent kinases
E2F: E2F transcription factors
KU70: XRCC6-binding protein 1
KU80: XRCC5-binding protein 1
HSP90: Heat shock protein 90
